# Obituary

**Published:** 1846-04-01

**Authors:** 


*Obituary.*—Died at Detroit, Mich., on the 11th ult., Lieut. E. R. Long, M. D., U. S. Army.

Justice to our own feelings and to the feelings of many of our readers, claims some tribute to the memory of our departed friend, whose decease it is our painful duty to record. Having been a resident in this city for several years previous to the present winter, he has left a large circle of attached friends, who mourn his loss with a greater depth of sorrow than ordinarily appertains to the sundering of the ties of friendship by the fell destroyer. Unassuming and gentle in disposition, scrupulously regardful of the opinions and feelings of others, without the least ostentation or affectation, possessing extensive and solid acquirements, united with a refined taste, he instinctively, as it were, won the respect and affection of all who knew him. We doubt if he ever had an enemy, or fell under the imputation of aught which would appear inconsistent with the most exact principles of justice and honor. They, however, who were fortunate enough to possess his intimacy can best appreciate the loss which not alone his kindred and friends, but society has sustained. His life was an exemplification of the domestic, social and christian virtues. These diffused around him a genial influence, while his silent example invited and encouraged imitation.

The medical profession have much cause for regret in his untimely death. Being graduated at West Point, he had been connected with the army for about fifteen years. The profession of arms, however, did not satisfy his wishes. Having a predilection for medicine, he was a diligent votary of its several departments for the last six years of his life. The winter of 1844–5 he passed at Chicago in attendance upon the medical lectures in that city, and received at their close the degree of M. D. As a characteristic instance of his faithful assiduity, it deserves to be stated that of all the lectures in the several departments (from 5 to 7 daily for a period of four months,) he lost his attendance upon a single one only, and this was accidental! His intention was ultimately to have relinquished his commission, and devote himself to his new profession. He had selected especially the department of medical chemistry, and gave every promise of future usefulness and eminence in that interesting and important branch. Our readers probably need not be reminded that several valuable articles from his pen are contained in the former numbers of this Journal. His health had for many years been delicate, but the disease that proved fatal was typhoid fever.

